# Draft Genome Sequence of a Mucoid Isolate of *Pseudomonas aeruginosa* Strain C7447m from a Patient with Cystic Fibrosis

**DOI:** 10.1128/genomeA.00837-13

**Published:** 2013-10-10

**Authors:** Yeshi Yin, T. Ryan Withers, Shannon L. Johnson, Hongwei D. Yu

**Affiliations:** Department of Biochemistry and Microbiology, Joan C. Edwards School of Medicine at Marshall University, Huntington, West Virginia, USAa; Department of Pediatrics, Joan C. Edwards School of Medicine at Marshall University, Huntington, West Virginia, USAb; Progenesis Technologies, LLC, Huntington, West Virginia, USAc; Genome Science Group (B6), Los Alamos National Laboratory, Los Alamos, New Mexico, USAd

## Abstract

Alginate overproduction by *Pseudomonas aeruginosa*, or mucoidy, plays an important role in the pathogenesis of chronic lung infections in cystic fibrosis (CF) patients. Here we report the draft genome sequence of a clinical isolate of mucoid *P. aeruginosa* strain C7447m from a CF patient with chronic lung infection.

## GENOME ANNOUNCEMENT

Overproduction of the capsular polysaccharide alginate by the Gram-negative bacterium *Pseudomonas aeruginosa* is a hallmark of chronic lung infections in cystic fibrosis (CF) patients. Among its many roles in virulence, alginate protects the bacteria from host defenses (1) and antibiotic chemotherapy (2). Here, we report the genome sequence of a clinical alginate-overproducing, mucoid *P. aeruginosa* isolate, C7447m. Strain C7447m was originally isolated from a CF patient in 1997 by David P. Speert in Vancouver, British Columbia, Canada.

Genomic DNA extracted by cetyltrimethylammonium bromide (CTAB)-NaCl and phenol-chloroform-isoamyl alcohol was sent to Cofactor Genomics (St. Louis, MO). Paired-end sequencing libraries were generated according to vendor protocols (Illumina, San Diego, CA). The genome sequencing was performed on an Illumina GAIIX. Totals of 11,770,660 raw reads and 1,883,305,600 bp were obtained. The sequence data were generated and assembled using Illumina Pipeline version SCS 2.8.0 based on paired-end tags with OLB 1.8.0. The sequences were then aligned and annotated according to the reference strain PAO1 genome (GenBank accession no. NC_2516.2) by use of the Novocraft novoAlign v 2.07.13 software package. Further analysis of the genome was performed using Samtools version 01.15c for the generation of pileup after sorting and the removal of duplicate reads. The analysis pipeline software was developed by CoFactor Genomics, and all specifics regarding aligner algorithms can be obtained from Novocraft Technologies. In summary, the number of generated base pairs resulted in approximately 224× coverage of the reference PAO1 genome. The number of base pairs saturated at or above 8× is 6,088,140 (97.19%), and the number of base pairs saturated below 8× is 176,264 (2.81%). The genome was annotated and prepared for submission using Ergatis-based workflow with manual correction.

The analysis results of single nucleotide polymorphisms (SNPs) and indels showed that 643 heterozygous mutants (255 indels and 388 SNPs; count ratios of ≥0.4 and ≤0.6) and 25,753 homozygous SNPs (count ratio, >0.6) were found. The count ratio is defined as the number of times the reference base is observed divided by the coverage at this base (counting all matches and mismatches). Only 14 indels, with coverage at the site above 8× and the mutant to wild type ratios at >2, were included in this genome edition. Thirteen of the included indels were distributed in the intergenic region. Among the homozygous SNPs, 22,439 mutations were distributed in 4,516 genes/coding sequence (CDS) (which accounts for 79.47% of the total genes/CDS), and 3,314 mutations were identified in the intergenic region. Most of the mutant genes have multiple SNPs, and 2,846 genes/CDS have 3 or more homozygous SNPs. Despite the mucoid phenotype, the strain C7447m has wild-type *algU* and *mucA*. Furthermore, among previously reported alginate production-related genes (3–6), the amino acid sequences of MucC, MucD, AlgL, and KinB each have 1 amino acid change; AlgX has 2 amino acid changes; and AlgI and AlgP have 5 amino acid changes. We also found some SNPs located in the intergenic region before *algB*, *algC*, *algD*, *amrZ*, *clpP2*, *mucE*, *mucR*, and *pilA*.

### Nucleotide sequence accession number.

The draft genome sequence of C7447m has been deposited in GenBank with the accession number CP006728.
